# Application of magnetic resonance diffusion kurtosis imaging for distinguishing histopathologic subtypes and grades of rectal carcinoma

**DOI:** 10.1186/s40644-019-0192-x

**Published:** 2019-02-11

**Authors:** Ziqiang Wen, Yan Chen, Xinyue Yang, Baolan Lu, Yiyan Liu, Bingqi Shen, Shenping Yu

**Affiliations:** 1 Department of Radiology, Sun Yat-sen University First Affiliated Hospital, No. 58, Zhongshan Second Road, Yuexiu District, Guangzhou, 510080 China; 2 Department of Radiology, Southern Medical University Zhujiang Hospital, Guangzhou, 510282 China

**Keywords:** Rectal cancer, Magnetic resonance imaging, Diffusion kurtosis imaging, Mucinous carcinoma, Imaging biomarker

## Abstract

**Background:**

To evaluate the diagnostic performance of diffusion kurtosis imaging (DKI) for distinguishing different histopathological subtypes and grades of rectal carcinoma and to compare DKI with conventional diffusion-weighted imaging (DWI).

**Methods:**

This prospective study involved 132 patients with rectal carcinoma, comprising 116 with adenocarcinoma not otherwise specified (AC) and 16 with mucinous carcinoma (MC). High spatial resolution magnetic resonance (MR) and DKI sequences (b values of 0, 600, 1000, 1500 and 2000 s/mm^2^) were performed for pretreatment evaluation. The mean kurtosis (MK) and mean diffusivity (MD) from DKI and the apparent diffusion coefficient (ADC) from DWI were measured by two experienced radiologists. The Mann-Whitney U test was used to evaluate different histopathological subtypes and grades. Receiver operating characteristic (ROC) curve analyses were performed to compare the diagnostic ability of different quantitative parameters.

**Results:**

The MD and ADC values were significantly higher for MC than for AC (1.94 ± 0.51 vs. 1.33 ± 0.02 and 1.26 ± 0.64 vs. 0.92 ± 0.01, respectively; *P* < 0.001). The MK values were significantly lower for MC than for AC (0.66 ± 0.02 vs. 0.93 ± 0.09, *P* < 0.001). The MK and MD values demonstrated higher sensitivity (94%, both) and specificity (96, 93%, respectively) than the ADC values. However, all the parameters derived from both DKI and DWI showed no significant differences between different histological grades.

**Conclusions:**

DKI is a more valuable imaging biomarker than conventional DWI for differentiating MC from AC. However, it is still debatable whether DKI is useful for distinguishing different histological grades.

## Background

Rectal carcinoma is one of the most common digestive malignant tumors worldwide [1], and for the past several years, evidence has revealed that the popularity of screening and changes in treatment strategies have delayed the progression of disease and decreased the overall mortality rates of patients with rectal carcinoma [1]. Previously, studies indicated that preoperative neoadjuvant therapy was effective for decreasing local recurrence and increasing the chance of enhancing sphincter preservation compared with surgery alone [2, 3]. However, the response to treatment in rectal carcinoma is associated with the histopathological subtype and histological grade of disease [4, 5]. In particular, mucinous carcinoma (MC) does not respond well to neoadjuvant therapy and has a poorer prognosis than other types of rectal carcinoma [6]. In addition, side effects from preoperative chemoradiotherapy are not uncommon [7]. Therefore, it is of considerable importance to assess related factors to determine individual treatment strategies and prevent side effects from unnecessary therapy.

High spatial resolution magnetic resonance (MR) imaging has been routinely used for the preoperative evaluation of rectal carcinoma [8–10]. In addition, functional MR imaging, such as diffusion-weighted imaging (DWI), has been widely regarded as a promising tool for preoperative assessment of rectal carcinoma. This imaging modality can provide information on a molecular level in addition to anatomical information [11–14]. However, conventional DWI assumes that the motion of water molecules distributes according to Gaussian behavior. Notably, the microenvironment in biological tissue is complex [15]. Water molecule diffusion with non-Gaussian behavior results from microstructures such as the cytomembrane and extracellular matrix and contributes to diffusion. Jensen et al. proposed a non-Gaussian diffusion model called diffusion kurtosis imaging (DKI), which includes mean kurtosis (MK) and mean diffusivity (MD), to quantify this deviation from Gaussian form [16]. In previous studies, DKI exhibited advantages for glioma, breast cancer and masses located in the head and neck region [17–20]. To our knowledge, there are few studies on DKI for differentiating histopathological subtypes and grades of rectal carcinoma, especially for MC.

Therefore, our study was aimed at proving the applicability of DKI in rectal carcinoma. We compared the features of adenocarcinoma not otherwise specified (AC), which was the most common form of rectal carcinoma and other special subtypes were excluded, and unaffected rectal wall tissues first and further compared parameters from DKI and DWI between different histologic subtypes and grades of rectal carcinoma.

## Methods

### Patients

**Fig. 1:**
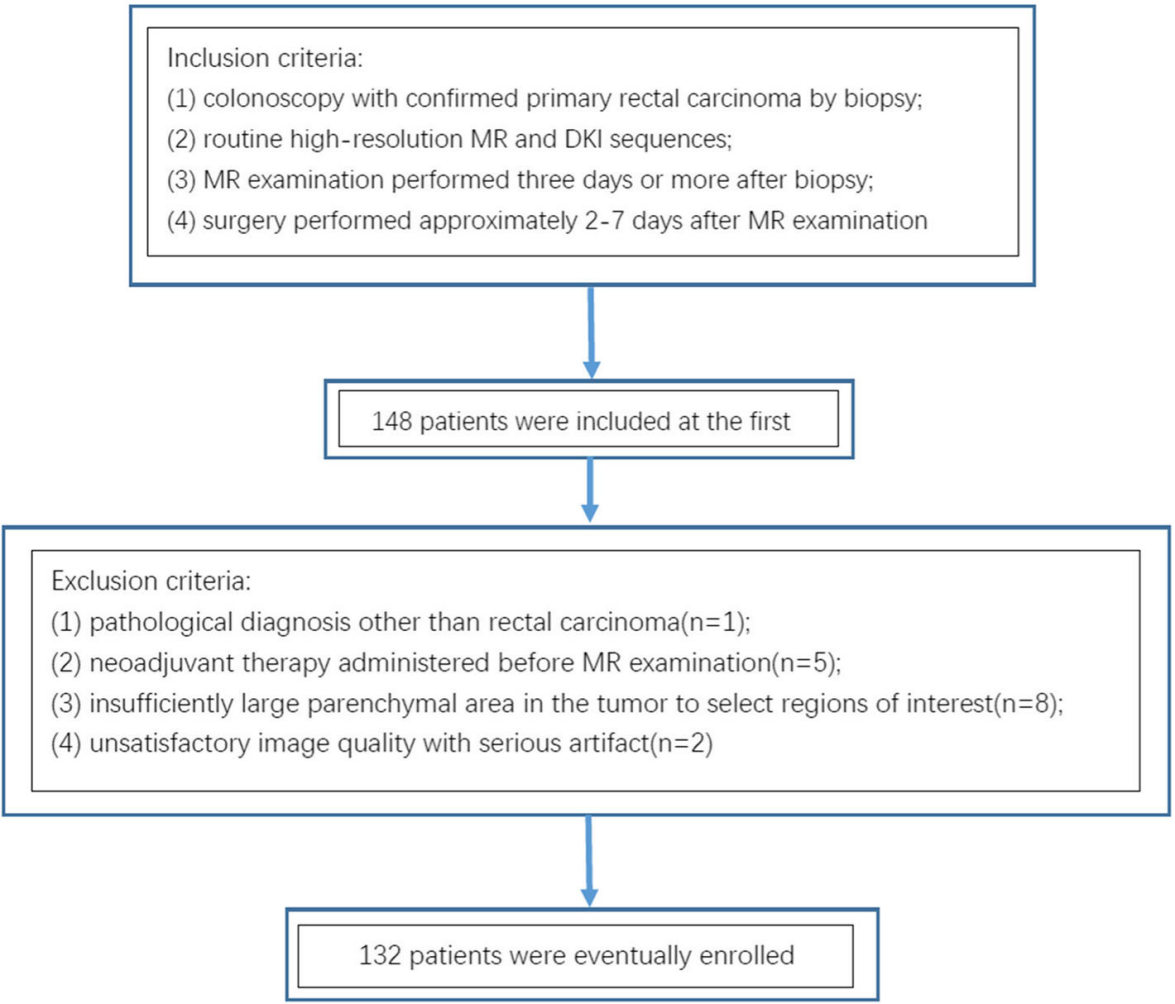
Flowchart of the study population

This prospective study was approved by our institutional ethics review board, and informed consent was obtained from all patients. The inclusion criteria were as follows: (1) colonoscopy with confirmed primary rectal carcinoma by biopsy; (2) routine high-resolution MR and DKI sequences; (3) MR examination performed three days or more after biopsy; and (4) surgery performed approximately 2–7 days after MR examination. So 148 patients were included in the study at the first. 16 patients were excluded for satisfying one of the following exclusion criteria: (1) pathological diagnosis other than rectal carcinoma; (2) neoadjuvant therapy administered before MR examination; (3) insufficiently large parenchymal area in the tumor to select regions of interest (ROIs); and (4) unsatisfactory image quality with serious artifact. Eventually 132 patients including 116 patients (median age, 52 years; range, 29–83 years; 70 men and 46 women) with AC and 16 patients (median age, 51 years; range, 22–67 years; 8 men and 8 women) with MC were enrolled in this prospective study (Fig. 1).

### MR examination

The patients in this study all underwent MR examinations with a 3.0-T MR scanner (Magnetom Verio, Siemens Healthcare, Erlangen, Germany) with a six-channel body matrix coil. Prior to the MR scan, 20–80 mL of ultrasound gel was injected into the rectum to display the boundaries of the tumor more clearly. The quantity of the gel injected into the rectum in each patient depended on the distance from the inferior part of the tumor to the anal verge. Each patient received a routine intramuscular injection of 20 mg of anisodamine to reduce artifacts caused by peristalsis of the intestinal tract. Gadolinium (Gadopentetate Dimeglumine Injection, Consun, Guangzhou, China) was intravenously injected using a power injector (Medrad, Pittsburgh, PA, USA) at 0.2 mL/kg of body weight at a rate of 3.0 mL/s.

Conventional rectal MR imaging included the following sequences: (1) an axial T2-weighted turbo spin-echo sequence with thick sections; (2) a high-resolution sagittal T2-weighted turbo spin-echo sequence with thin sections in sagittal, coronal and oblique axial planes.

A DKI sequence was performed in an axial plane through the use of a single-shot echo-planar imaging sequence with b values of 0, 600, 1000, 1500 and 2000 s/mm^2^ (Table 1).

**Table 1:** MR Imaging Protocol Parameters for T2-Weighted Imaging and DKI

	Imaging Sequence				
		Sagittal	Coronal	Oblique Axial	Axial
	Axial	High-resolution	High-resolution	High-resolution	Diffusion-
Parameter	T2W	T2W	T2W	T2W	kurtosis
Pulse sequence	TSE	TSE	TSE	TSE	DKI-EPI
TR/TE (ms)	3000/87	3000/87	4000/77	3000/84	3800/74.7
Echo train length	14	12	21	16	/
FOV (mm^2^)	260 × 260	180 × 180	220 × 220	180 × 180	300 × 245
Section thickness (mm)	5	3	3	3	6
No. of slices	25	19	25	24	21
Dist. factor	20%	0	0	0	20%
Phase oversampling	70%	70%	100%	70%	20%
Voxel size (mm^3^)	0.8 × 0.7 × 5.0	0.7 × 0.6 × 3.0	0.7 × 0.6 × 3.0	0.7 × 0.6 × 3.0	2.7 × 2.7 × 6.0
Averages	2	2	2	2	2
Concatenation	2	2	1	2	1
Acquisition time	2 min 56 s	2 min 30 s	2 min 52 s	3 min 18 s	6 min 1 s

Note: *DKI* = diffusion kurtosis imaging, *EPI* = echo-planar imaging, *TSE* = turbo spin-echo, T2W = T2-weighted, TR = repetition time, TE = echo time, FOV = field of view

### Imaging analysis

All primary data of 132 patients were uploaded to a workstation, and parameter maps of DKI were generated by using postprocessing software that was developed in house (MATLAB Version 2.1; MathWorks, Natick, MA, USA). For the DWI dataset, imaging data of 2 b values (0, 1000s/mm^2^) were processed to calculate ADC maps. Data of 5 b values (0, 600, 1000, 1500 and 2000 s/mm^2^) were processed using a three-variable linear least-squares method based on a DKI model similar to that implemented in a previous study to get MK and MD maps [21].The linear fitting equation is as follows:

$$ 1\mathrm{n}\left({S}_b\right)=1\mathrm{n}\left({S}_0\right)-\mathrm{b}\cdot \mathrm{D}+1/6\cdot {b}^2\cdot {D}^2\cdot K $$

where S_b_ indicates the MR signal intensity at the particular b value used; S_0_ indicates the MR signal intensity when there is no diffusion gradient; K is the apparent diffusional kurtosis, which is a dimensionless metric of departure from Gaussian behavior of water motion; and D is the apparent diffusion coefficient (ADC), which has been revised for non-Gaussian behavior.

**Fig. 2:**
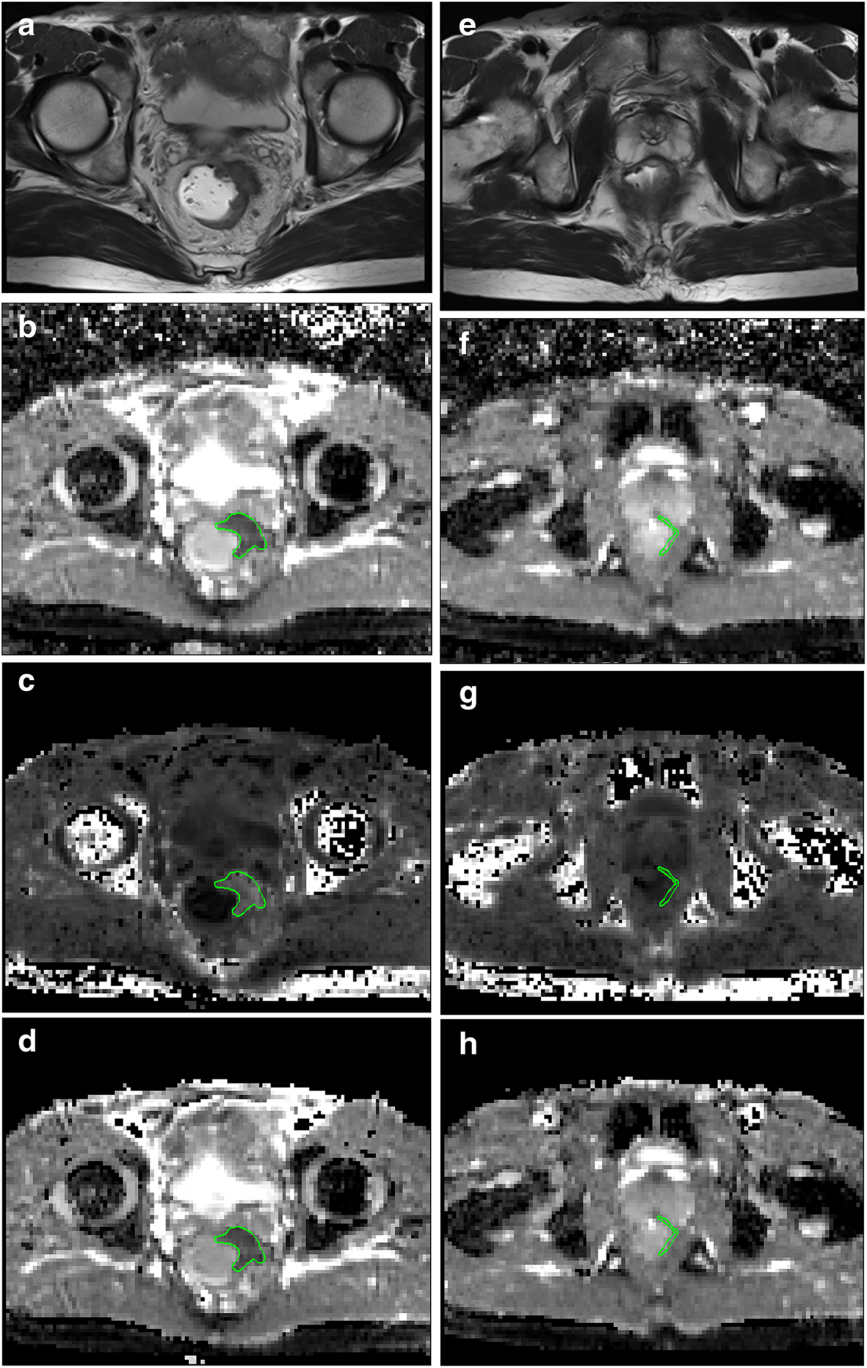
Images above show Grade 1 AC and unaffected rectal wall in a 62-year-old man. Axial T2-weighted MR image shows the AC along the left wall of the rectum (**a**). ADC map shows low-signal-intensity tumor (ADC = 1.09 ± 0.24 × 10^− 3^ mm^2^/s, **b**). MK map shows high-signal-intensity tumor (MK = 1.00 ± 0.19, **c**). MD map shows low-signal-intensity tumor (MD = 1.26 ± 0.24 × 10^− 3^ mm^2^/s, **d**). Axial T2-weighted MR image shows the unaffected rectal wall (**e**). ADC map shows high-signal-intensity rectal wall (ADC = 1.69 ± 0.10 × 18^− 3^ mm^2^/s, **f**). MK map shows low-signal-intensity rectal wall (MK = 0.57 ± 0.10, **g**). MD map shows high-signal-intensity rectal wall (MD = 2.04 ± 0.21 × 10^− 3^ mm^2^/s, h)

**Fig. 3:**
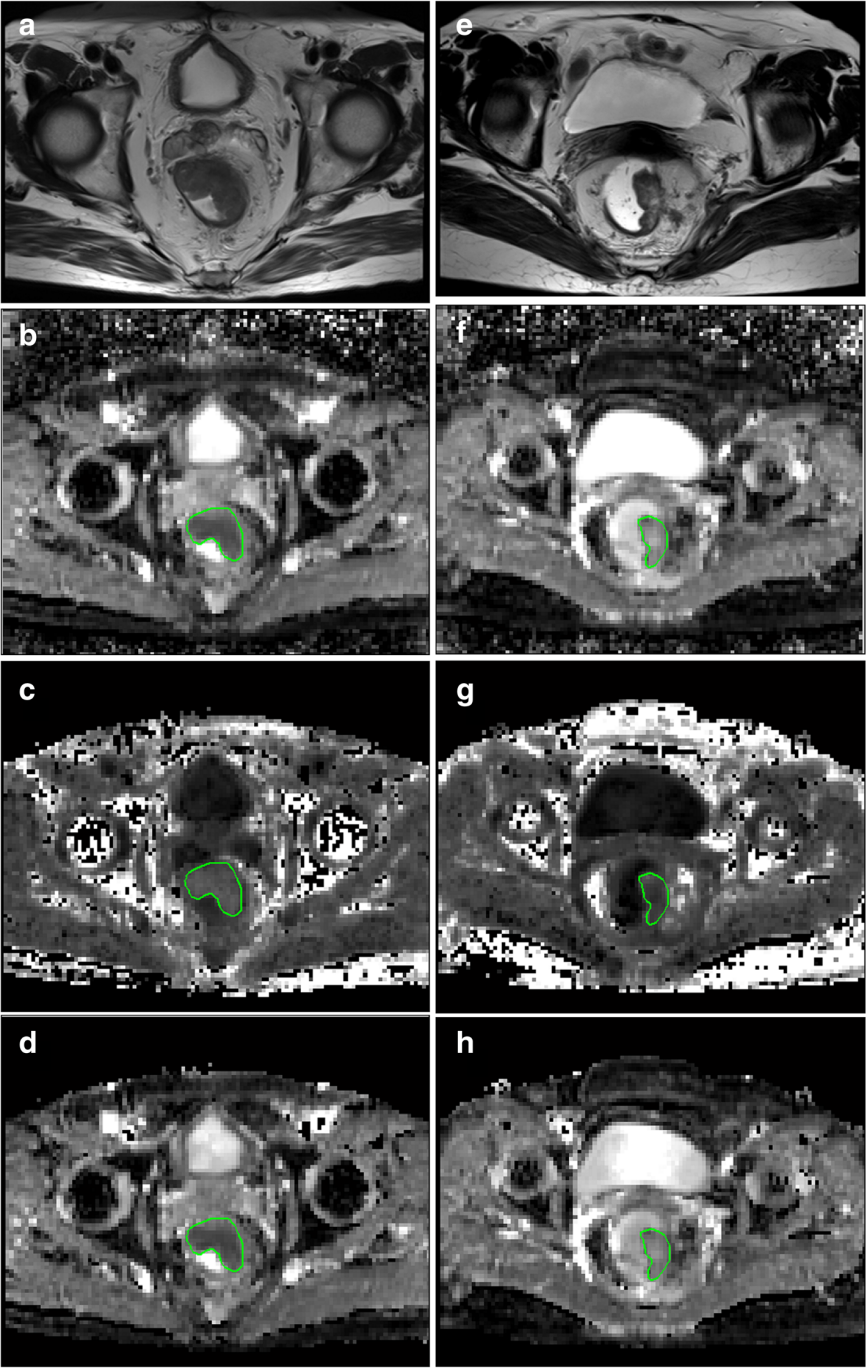
Images above show a 54-year-old man with Grade 2 AC (**a**-**d**) and a 72-year-old woman with Grade 3 MC (**e**-**h**). Axial T2-weighted MR image shows the AC along the anterior and left wall of the rectum (**a**). ADC map shows low-signal-intensity tumor (ADC = 1.21 ± 0.92 × 10^− 3^ mm^2^/s, **b**). MK map shows high-signal-intensity tumor (MK = 1.02 ± 0.01, **c**). MD map shows low-signal-intensity tumor (MD = 1.19 ± 0.21 × 10^− 3^ mm^2^/s, **d**). Axial T2-weighted MR image shows the MC along left wall of the rectum (**e**). ADC map shows high-signal-intensity tumor (ADC = 1.84 ± 0.57 × 10^− 3^ mm^2^/s, **f**). MK map shows low-signal-intensity tumor (MK = 0.72 ± 0.15, **g**). MD map shows high-signal-intensity tumor (MD = 1.88 ± 0.31 × 10^− 3^ mm^2^/s, h)

The patients’ MR data were reviewed by two seasoned radiologists who were blinded to the pathological and clinical information. The three largest sections of tumor parenchyma and three sections of unaffected rectal wall that were located more than 1 cm from the tumor were manually drawn on high-b-value images and later were copied that to all DWI and DKI derived maps (Figs. 2, 3). The ROIs were confirmed by comparing the ROI position in the parameter maps to axial T2-weighted imaging. Areas of necrosis, cystic degeneration and intestinal contents were avoided. The pixel-based mean value of the three sections represented the value of the tumor and unaffected rectal wall, which were averaged for comparison. The values measured from radiologist 1 were used in comparison.

### Pathological assessment

All the surgical specimens were routinely processed by the pathology department with fixation in formalin and stained with hematoxylin-eosin. Pathological evaluation was performed by pathologists on the basis of the seventh edition of the American Joint Committee on Cancer’s TNM classification [22]. Tumors were classified as Grade 1 (well differentiated) when gland-like structures accounted for more than 95% of the specimen, Grade 2 (moderately differentiated) when these structures occupied more than 50% but less than or equal to 95% of the specimen, or Grade 3 (poorly differentiated) when these structures occupied less than or equal to 50% of the specimen. MC was characterized by abundant extracellular mucin that constituted more than 50% of the tumor volume [22].

### Statistical analysis

The numeric data of DKI and DWI parameters measured by the two radiologists were divided into different groups. The mean and standard deviation were calculated for each parameter. The Kolmogorov-Smirnov test was used to perform a normality analysis on the data of the different groups. The data were not normally distributed. So the Wilcoxon test was used to compare the differences in each parameter in the following groups: AC and unaffected rectal wall. The differences in each parameter in these groups (MC vs. AC; Grade 1–2 vs. Grade 3) were assessed by using the Mann-Whitney U test. Receiver operating characteristic (ROC) curve analysis was performed to assess the diagnostic performance of each parameter. Sensitivity and specificity were measured with a threshold determined by using the maximum Youden’s index. Area under the curves (AUC) were compared using the method developed by Delong et al. [23]

Interreader agreement for DKI and DWI derived parameter measurements were estimated by using interclass correlation coefficient (ICC) and were interpreted as follows: ICC > 0.75 indicated excellent agreement, 0.60–0.75 indicated good agreement, 0.40–0.60 indicated fair agreement, and less than 0.40 indicated poor agreement. Statistical analysis was performed with SPSS (version 23.0) and MedCalc (version 15.8) software. *P* values< 0.05 were considered statistically significant.

## Results

### Patient demographics and histological results

In total, 132 patients satisfied the study criteria mentioned above, and ultimately 116 patients (70 men and 46 women; median age: 52 years; age range: 29–83 years) with AC and 16 patients (8 men and 8 women; median age: 51 years; age range: 22–67 years) with MC were enrolled.

The pathological evaluation by using the WHO grading criteria was as follows: AC (Grade 1: *n* = 1; Grade 2: *n* = 93; Grade 3: *n* = 22) and MC (Grade 1: *n* = 1; Grade 2: n = 9; Grade 3: *n* = 6). The correlative pathological and clinical characteristics are listed in Table 2.

**Table 2:** Clinical and Pathological Characteristics

	AC (116)	MC (16)
Age		
Median age	52	51
Age range	29–83	22–67
Gender		
Male	70	8
Female	46	8
Histological Grade		
Grade 1	1	1
Grade 2	93	9
Grade 3	22	6
MR T Stage		
T1	0	0
T2	17	2
T3	59	9
T4	40	5

Note: *AC* = adenocarcinoma not otherwise specified, *MC* = mucinous carcinoma

### Interobserver agreement

The interobserver agreement between the two radiologists was assessed by the ICC (Table 3). There was excellent agreement between the two observers for MK, MD, and ADC.

**Table 3:** ICC for the Parameters Measured by Two Observers

Parameters	MK	MD	ADC
ICC (95% CI)	0.96 (0.93, 0.97)	0.97 (0.96, 0.98)	0.78 (0.69, 0.85)

Note: Data in parentheses are 95% confidence intervals, *ICC* = interclass correlation coefficient, *MK* = mean kurtosis, *MD* = mean diffusivity, *ADC* = apparent diffusion coefficient

### Comparison between AC and unaffected rectal wall

**Fig. 4:**
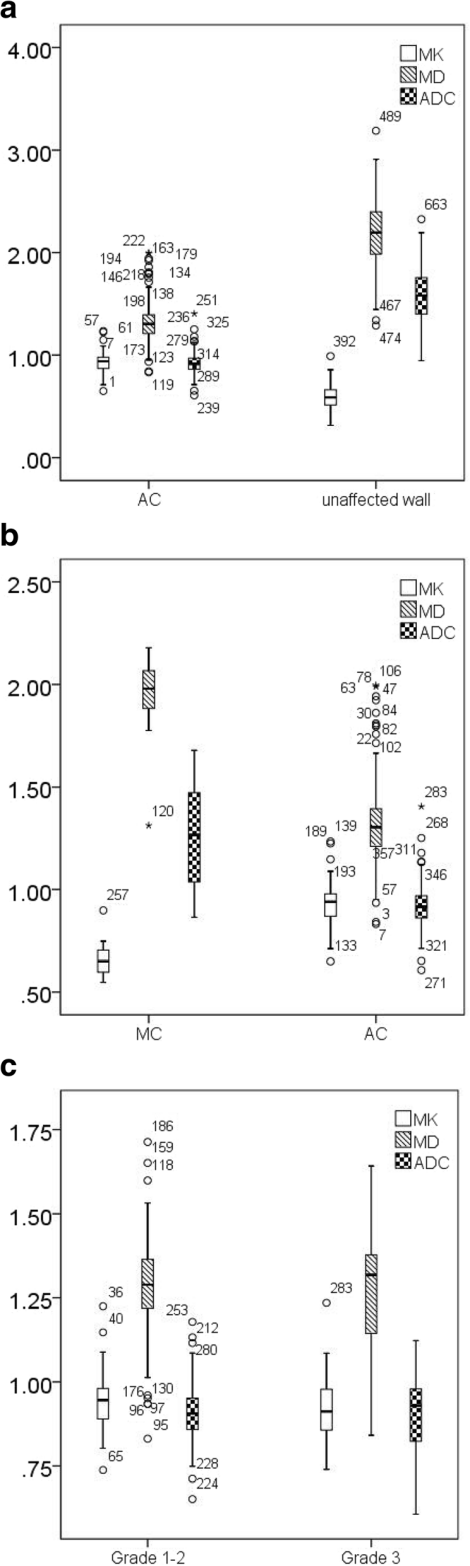
Box plots showing comparison of MK, MD and ADC among different groups (**a**, AC vs. unaffected wall; **b**, MC vs. AC; **c**, Grade 1–2 AC vs. Grade 3 AC)

The MD and ADC values were significantly lower in AC than in unaffected rectal wall (1.33 ± 0.02 vs. 2.20 ± 0.34, *P* < 0.001 for MD; 0.92 ± 0.01 vs. 1.59 ± 0.03, *P* < 0.001 for ADC). The MK values for AC were significantly higher than those for unaffected rectal wall (0.93 ± 0.09 vs. 0.59 ± 0.12, *P* < 0.001) (Table 4) (Fig. 4a).

**Table 4:** MK, MD and ADC values Among Different Groups

	AC	Unaffected wall	MC	AC	Grade 1–2 AC	Grade 3 AC
Number	116	116	16	116	94	22
MK	0.93 ± 0.09	0.59 ± 0.12	0.66 ± 0.02	0.93 ± 0.09	0.94 ± 0.08	0.92 ± 0.12
*P*	< 0.001		< 0.001		0.22	
MD	1.33 ± 0.02	2.20 ± 0.34	1.94 ± 0.51	1.33 ± 0.02	1.28 ± 0.14	1.28 ± 0.18
*P*	< 0.001		< 0.001		0.97	
ADC	0.92 ± 0.01	1.59 ± 0.03	1.26 ± 0.64	0.92 ± 0.01	0.91 ± 0.83	0.90 ± 0.12
*P*	< 0.001		< 0.001		0.90	

Note: Data are means ± standard deviations, MD and ADC values are given in mm^2^/s × 10^−3^, AC = adenocarcinoma not otherwise specified, MC = mucinous carcinoma, MK = mean kurtosis, MD = mean diffusivity

### Performance of MK, MD and ADC for distinguishing MC from AC

**Fig. 5:**
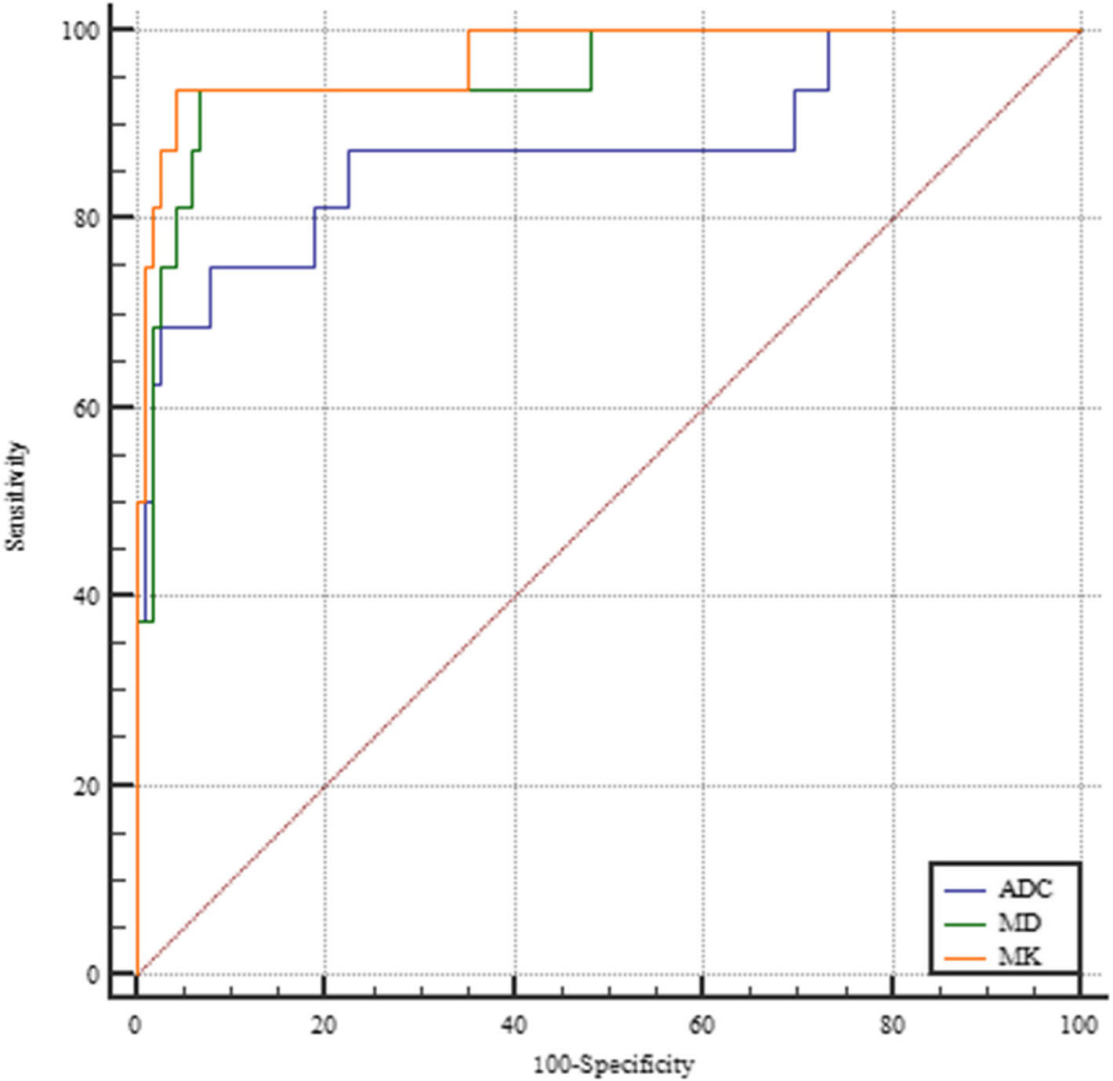
ROC curves of performance of MK, MD and ADC for discriminating MC from AC

The MD and ADC values were significantly higher in MC than in AC (1.94 ± 0.51 vs. 1.33 ± 0.02, *P* < 0.001 for MD; 1.26 ± 0.64 vs. 0.92 ± 0.01, *P* < 0.001 for ADC). The MK values were significantly lower in MC than in AC (0.66 ± 0.02 vs. 0.93 ± 0.09, *P* < 0.001) (Table 4) (Fig. 4b). ROC analysis demonstrated that MK showed the highest sensitivity (94%) and specificity (96%) with a cut-off value of 0.75 (Table 5). Comparisons of the ROC curves of these parameters indicated that MK had the highest area under the curve (AUC) of 0.97. There was no significant difference between the AUC derived from DKI and conventional DWI. However, the AUC of MK and MD were higher than that of ADC substantially (Fig. 5).

**Table 5:** Receiver Operating Characteristic Analysis of MK, MD and ADC

Parameters	MK	MD	ADC
AUC	0.97	0.95	0.88
*P*_AUC_	MK vs. MD	MK vs. ADC	MD vs. ADC
	0.64	0.15	0.24
Cut-Off Value	≤0.75	> 1.76	> 1.06
Sensitivity (100%)	0.94	0.94	0.75
Specificity (100%)	0.96	0.93	0.92

Note: *AUC* = area under ROC curve, *ROC* = receiver operating characteristic

### Performance of MK, MD and ADC for distinguishing grade 1–2 AC from grade 3 AC

All the parameters showed no significant difference between Grade 1–2 and Grade 3 AC (Table 4) (Fig. 4c).

## Discussion

In our study, all the parameters derived from DKI and conventional DWI showed significant differences between AC and unaffected rectal wall. In addition, our study revealed another important finding; namely, MC showed higher MD and ADC values than those of AC. The MK values in MC were lower than those in AC. However, all the parameters were not different between Grade 1–2 AC and Grade 3 AC.

In agreement with previous study [24], the ADC values for unaffected rectal wall were significantly higher than those for AC. For the DKI derived parameters, the MD values for unaffected rectal wall were significantly higher than those for AC while the MK values were significantly lower for unaffected rectal wall. These differences in the parameters obtained from conventional DWI and DKI in AC may indicate the clinical feasibility of DKI for evaluating rectal carcinoma. The high cellularity and decreased extracellular space in these tumors may cause restriction of water molecular motion [25], which can explain the differences between tumor and unaffected rectal wall tissues.

MC is distinguished as a totally different subtype of colorectal carcinoma with a poor prognosis and poor response to preoperative chemoradiotherapy [6]. Accurate diagnosis of this subtype of rectal carcinoma is significant for determining an appropriate treatment plan. Yu et al. [26] found that preoperative MR imaging was a more accurate method for detecting MC than biopsy sampling. However, the T2-weighted MR evaluation is still a subjective method because its diagnostic accuracy is correlated with the experience of the interpreting radiologists. Thus, we hope to use functional MR imaging techniques, such as DKI and DWI, to quantitatively distinguish MC from AC. In our study, the MD and ADC values for MC were higher than those for AC; these findings are in agreement with those of Nasu et al. [27]. We assumed that these differences might be associated with the amount of extracellular mucus in mucinous rectal carcinoma, which causes low cellularity, while the higher cellularity in AC might further restrict water molecule movement in tumor tissue. On the other hand, our study indicated that the MK values were significantly lower in MC than in AC. As an extension of conventional DWI, DKI can reflect tissue heterogeneity [16]. Because of its high cellularity and various membranes, AC may lead to more deviation from Gaussian behavior. In addition, the results of the ROC analysis demonstrated that using the MK and MD values obtained from DKI might have higher specificity and sensitivity for distinguishing MC from AC, suggesting better diagnostic performance of DKI than conventional DWI. Similar findings have been reported in a previous study that showed the advantages of DKI in different diseases [18–20, 28]. These findings suggest that DKI can offer additional information regarding changes in microstructure in different tumor tissues.

Our study also suggested that there were no significant differences in any of the parameters for distinguishing Grade 1–2 and Grade 3 AC. Similar results have been observed in some previous studies [24]. However, our results were contrary to those of another study by Zhu L et al. [21]. We hypothesize that this difference might be caused by different selection of b values between the two studies because the number of b values used and the actual b values are crucial for optimal fitting DKI model [24]. On the other hand, the sample sizes might also have been an important factor that impacted the results. In our study, the number of Grade 1 AC was relatively few, which might have caused a bias. Therefore, the application of DKI and DWI for pathologic grading of rectal carcinoma remains debatable.

Additionally, the ICC of ADC values was lower substantially than that of MK and MD values relatively, which was incomprehensible because they were measured from the same ROI. However, similar finding also can be found in other previous study. According to Lambregts et al. [29] research, we assumed that single-slice evaluation might be one of the reason for this different ICC while whole-volume evaluation might provide more reproducible results.

Our study has several limitations. First, this was a single-center study, and this sample size was not large enough, especially for Grade 1 rectal carcinoma, which might have resulted in bias in the analysis of different grade groups. Thus, a multicenter study of a large cohort is necessary. Furthermore, single-slice ROI analysis might not have accurately reflected the characteristics of the whole tumor parenchyma. Histogram analysis could give more comprehensive information. Finally, injection of ultrasound gel into the rectum might have thinned the unaffected wall and increased the measurement error. A controlled test without injecting gel should be performed to determine the effect of this bias.

## Conclusions

DKI is a potentially promising technique for distinguishing MC from AC. Furthermore, parameters derived from DKI performed better than those derived from conventional DWI for differentiating between subtypes of rectal carcinoma. However, it is still debatable whether DKI or DWI can distinguish between different histopathological grades of rectal carcinoma.

## Abbreviations

AC: Adenocarcinoma not otherwise specified
ADC: Apparent diffusion coefficient
AUC: Area under curve
CI: Confidence interval
DKI: Diffusion kurtosis imaging
DWI: Diffusion-weighted imaging
ICC: Interclass correlation coefficient
MC: Mucinous carcinoma
MD: Mean diffusivity
MK: Mean kurtosis
MR: Magnetic resonance
ROC: Receiver operating characteristic
ROI: Region of interest
TA: Acquisition time
TE: Echo time
TR: Repetition time
